# RNA polymerase II-associated proteins reveal pathways affected in neural precursors with VCP mutation

**DOI:** 10.1093/brain/awad046

**Published:** 2023-06-01

**Authors:** Mahmoud-Reza Rafiee, Sara Rohban, Karen Davey, Jernej Ule, Nicholas M Luscombe

**Affiliations:** 1 The Francis Crick Institute, 1 Midland Road, London NW1 1AT, UK; 2 Department of Neuromuscular Diseases, UCL Queen Square Institute of Neurology, Queen Square, London, WC1N 3BG, UK; 3 UK Dementia Research Institute Centre, King’s College London, London, UK; 4 UCL Genetics Institute, University College London, Gower Street, London WC1E 6BT, UK; 5 Okinawa Institute of Science & Technology Graduate University, Okinawa 904-0495, Japan

**Keywords:** RNA polymerase II, SPACE, Proteomics, ALS, VCP

## Abstract

Valosin-Containing Protein (VCP) is a hexameric ATPase Associated with diverse cellular Activities. Genetic mutations in VCP are associated with several forms of muscular and neuronal degeneration, such as amyotrophic lateral sclerosis (ALS). However, little is known about the effects of ALS-associated VCP mutations on the transcriptional machinery. Here, we used silica particle-assisted chromatin enrichment and mass spectrometry (SPACE-MS) to study proteins co-localized with RNA polymerase II (RNAP2) in precursor neurons differentiated from ALS patients or control induced pluripotent stem (iPS) cells. Remarkably, we observed three perturbed pathways in the mutant cells: First, we found diminished binding of proteins involved in mRNA splicing and transcription elongation in the mutant cells. Interestingly, knockdown of SART3, a recycling factor of the splicing machinery, leads to perturbed intron retention in ALS-associated genes. Second, among the regulators of TDP-43 we found reduced levels of RBM45, EIF5A, and RNF220 whose mutations were reported to be associated with neurodegenerative disorders. Third, we observed increased levels of heat shock proteins in the RNAP2 complex of the mutant cells. Together, these findings shed light on how transcription and splicing machinery are impaired in an ALS model system with VCP mutations and provide novel insights into the initial events that might lead to proteinopathy and aberrant alternative splicing in neurodegeneration.

VCP (p97 or Cdc48) is a highly conserved member of the type II AAA+ (ATPase Associated with diverse cellular Activities) superfamily of proteins and is a central component of the ubiquitin protease system that is integral to cellular proteostasis ^1^. VCP plays crucial roles in multiple cellular processes, including protein degradation, intracellular trafficking, DNA repair and replication, transcription, and cell cycle regulation ^2^. On chromatin, VCP was shown to facilitate ubiquitin-driven segregation of several proteins from DNA ^3^. VCP directly interacts with RNAP2 and plays an important role in mediating RNAP2 degradation during DNA damage ^4, 5^ or unstable elongation ^6^. However, little is known about how mutations of VCP affect RNAP2 and the associated factors in neurodegenerative diseases.

In fact, VCP mutations were reported in a wide range of disorders including frontotemporal dementia, Charcot-marie-tooth disease, inclusion body myopathy, Paget disease of bone, Parkinson’s disease, and amyotrophic lateral sclerosis ^7^. While cytoplasmic accumulation and nuclear loss of TDP-43 in motor neurons are considered a hallmark of the ALS disease at late stages ^8^, abnormal alternative splicing is an earlier sign of the disease ^9^ which implies RNAP2 accessory proteins are probably involved in the onset of the disease. Previous studies have shown that alternative splicing has a crucial role in brain development ^10^, aging ^11^, and neurodegenerative disorders ^12^; however, the link between VCP mutations, RNAP2 accessory factors, aberrant alternative splicing, and TDP-43 proteinopathy is not clear.

Here, we studied RNAP2-associated proteins in ALS-patient-derived human iPS cells with VCP mutations undergoing neurogenesis. Using chromatin immunoprecipitation combined with SPACE-MS we compared patient and control neural precursors to identify deregulated proteins. Our results indicate that in the mutant cells several mRNA splicing factors including regulators of TDP-43 are depleted, while chaperones are upregulated in association with RNAP2. Our approach thus provides an insight into the perturbed pathways in ALS.

## Results

### Pathways involved in neurodegenerative disorders are enriched by RNAP2-associated factors

**Figure 1:**
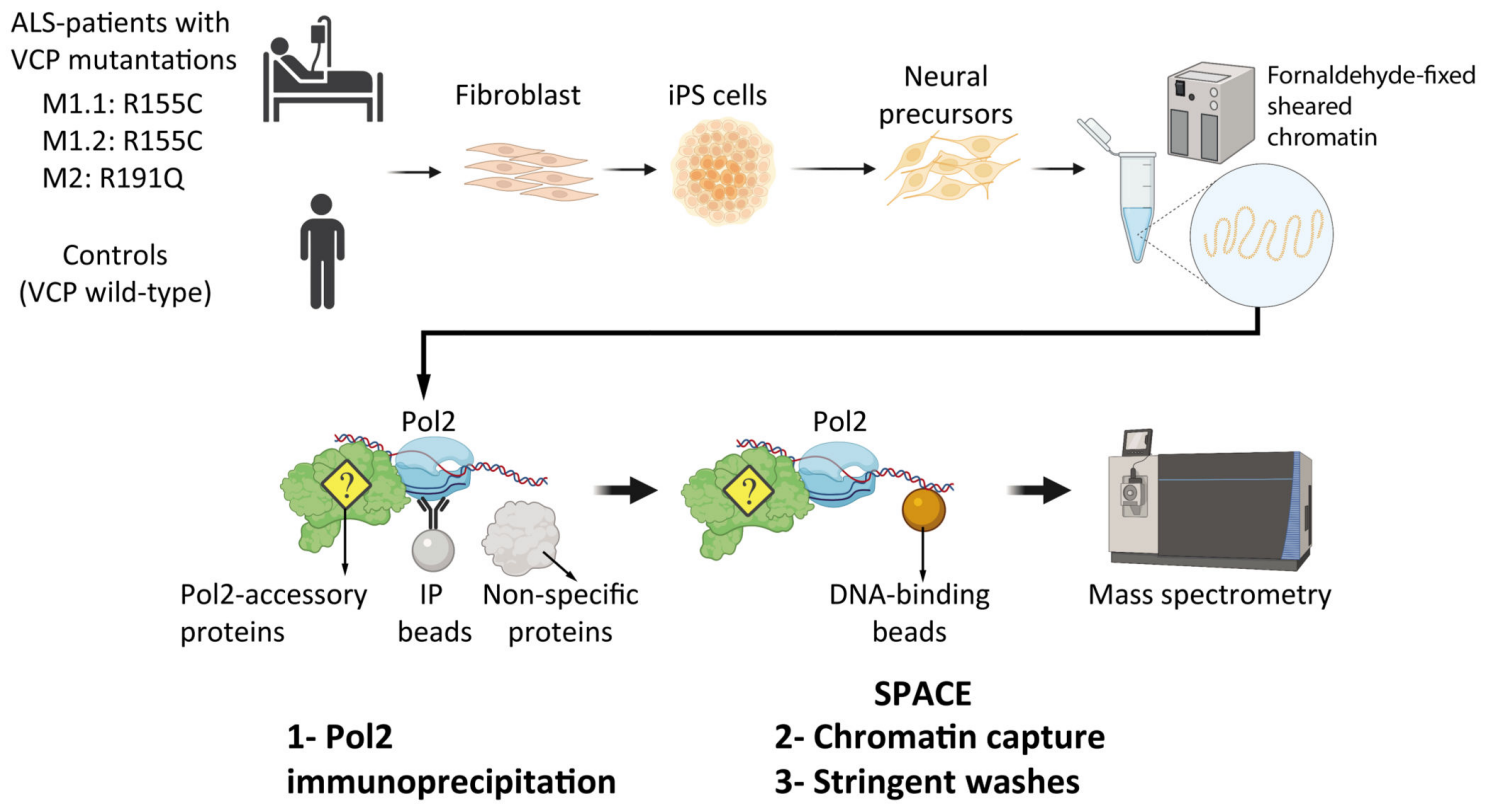
Schematic presentation of the experiments. Three ALS-patient iPS cell lines and three control iPS cell lines were differentiated to neural precursors until day 14. For each cell line, two replicates were used. The cells were fixed by formaldehyde and subjected to the ChIP procedure to fragment chromatin using sonication. After the immunoprecipitation by a pan-RNAP2 antibody, chromatin fragments were purified using silica magnetic DNA-binding beads (the SPACE procedure) to remove artificial interactions and to identify co-localized proteins with RNAP2.

**Figure 2:**
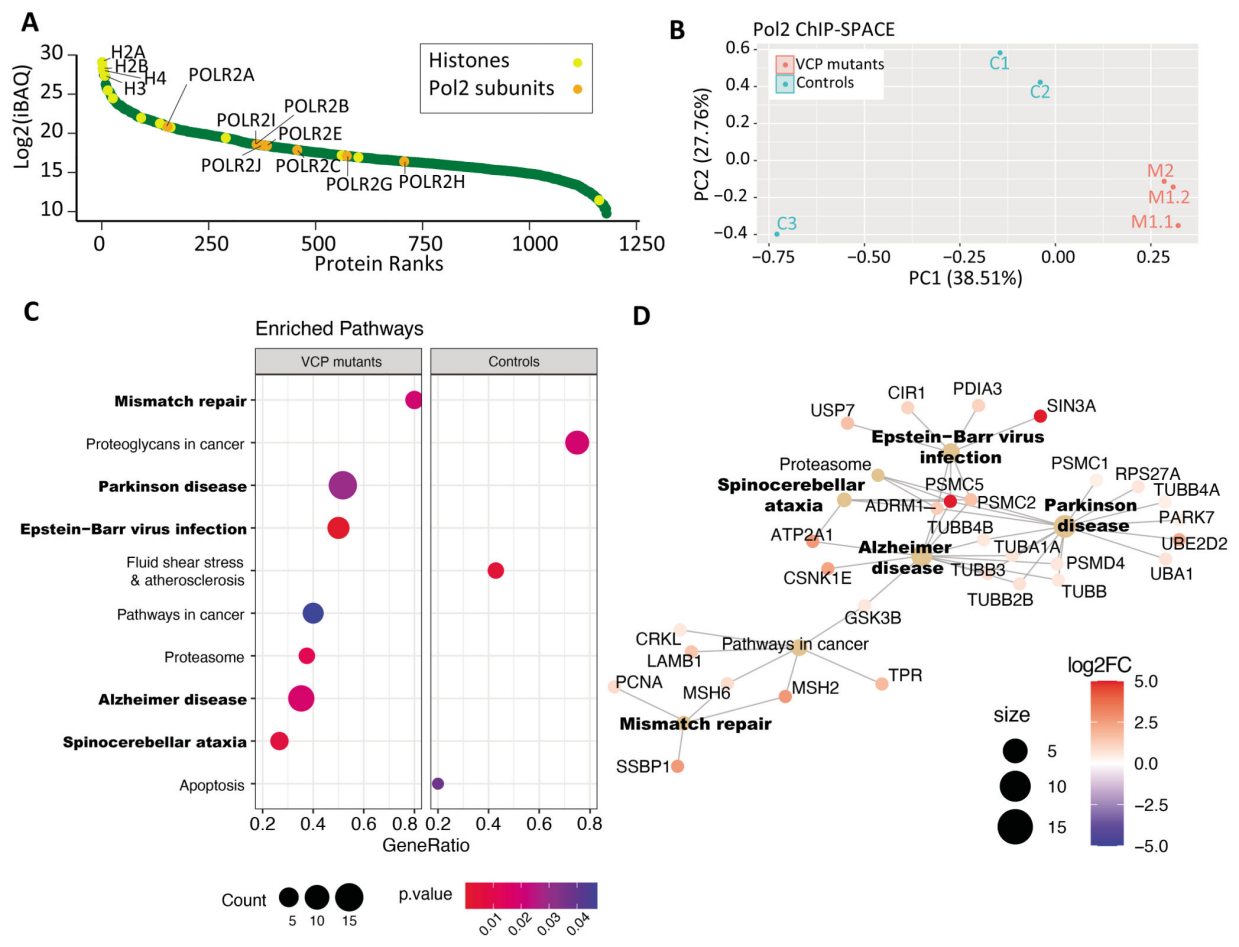
Co-localized proteins with RNAP2 in VCP mutant and control neural precursors. (A) The RNAP2-associated proteins were sorted based on their iBAQ values (normalized protein intensities). (B) Dimensionality reduction of the iBAQ intensities using PCA separates mutant and control cells using PC1. (C) KEGG pathway gene set enrichment analysis based on log2-fold-change ratios of the proteins in the mutant and control cells. Node size and color show number of genes involved in each pathway and p-value of enrichment, respectively. (D) The enriched pathway and their related genes were shown as a network. Node size and color show number of genes involved in each pathway and log2-fold-change of mutant/control, respectively.

To understand the potential effect of VCP mutations on RNAP2 function in neurodegeneration, we used human iPS cells obtained from ALS patients with heterozygous VCP mutantion which were previously used as cellular models of ALS phenotypes ^13^. The cells were differentiated to neuronal precursors to resemble a model of early events during neurodegeneration (Figure 1). Two R155C VCP mutant cell lines (M1.1 and M1.2 obtained from one patient) and one R191Q VCP mutant cell line (M2) were compared with three control cell lines from three healthy donors. We fixed the cells in the plates by adding formaldehyde to crosslink the chromatin-binding proteins to DNA. We then sheared chromatin to <1 kb fragments by sonication. We targeted RNAP2 by immunoprecipitation to identify the associated proteins. We then used DNA-binding beads (SPACE procedure) to stringently purify chromatin fragments and to remove artificial interactions that are produced during the immunoprecipitation. Following mass spectrometry, we compared the proteins identified by RNAP2 and normal IgG to remove the potential background proteins. After quantifying the proteins by mass spectrometry, we sorted the proteins based on their iBAQ values (Intensity Based Absolute Quantification) to check the quality of chromatin purification. Histones are the most abundant proteins, followed by RNAP2 components (Figure 2A). Comparing iBAQ values separated the control and mutant cells using Principal Component Analysis (Figure 2B). Interestingly, KEGG pathway enrichment by gene set enrichment analysis shows that neurodegenerative disorders such as Parkinson’s disease, Alzheimer’s disease and Spinocerebellar ataxia are enriched in the mutant cells (Figure 2 C-D). Additionally, we observed the enrichment of mismatch repair and Epstein-Barr virus (EBV) infection in the mutant cells. Increasing evidence has linked neurodegeneration to DNA damage and dysfunctional repair ^14^. Specifically, ALS-associated mutations were shown to affect DNA damage response activation. As such, DNA damage is accumulated in motor neurons differentiated from patients induced pluripotent stem cells and in spinal cord tissues ^15–17^. Recent studies have shown that EBV can induce neuroinflammation during latency and reactivation phases by arising systemic immune response, which leads to demyelination in Multiple Sclerosis ^18^ and neurodegeneration in Parkinson’s disease and Alzheimer’s disease ^19^. The underlying mechanism, however, is not understood and further mechanistic studies are required to elucidate the role of viral infection in the pathology of ALS. Altogether, this result indicates characterized and novel pathways involved in neurodegeneration and highlights the quality of the data for studying the effect of VCP mutations in ALS.

### ALS-associated proteins differentially bind RNA RNAP2

**Figure 3:**
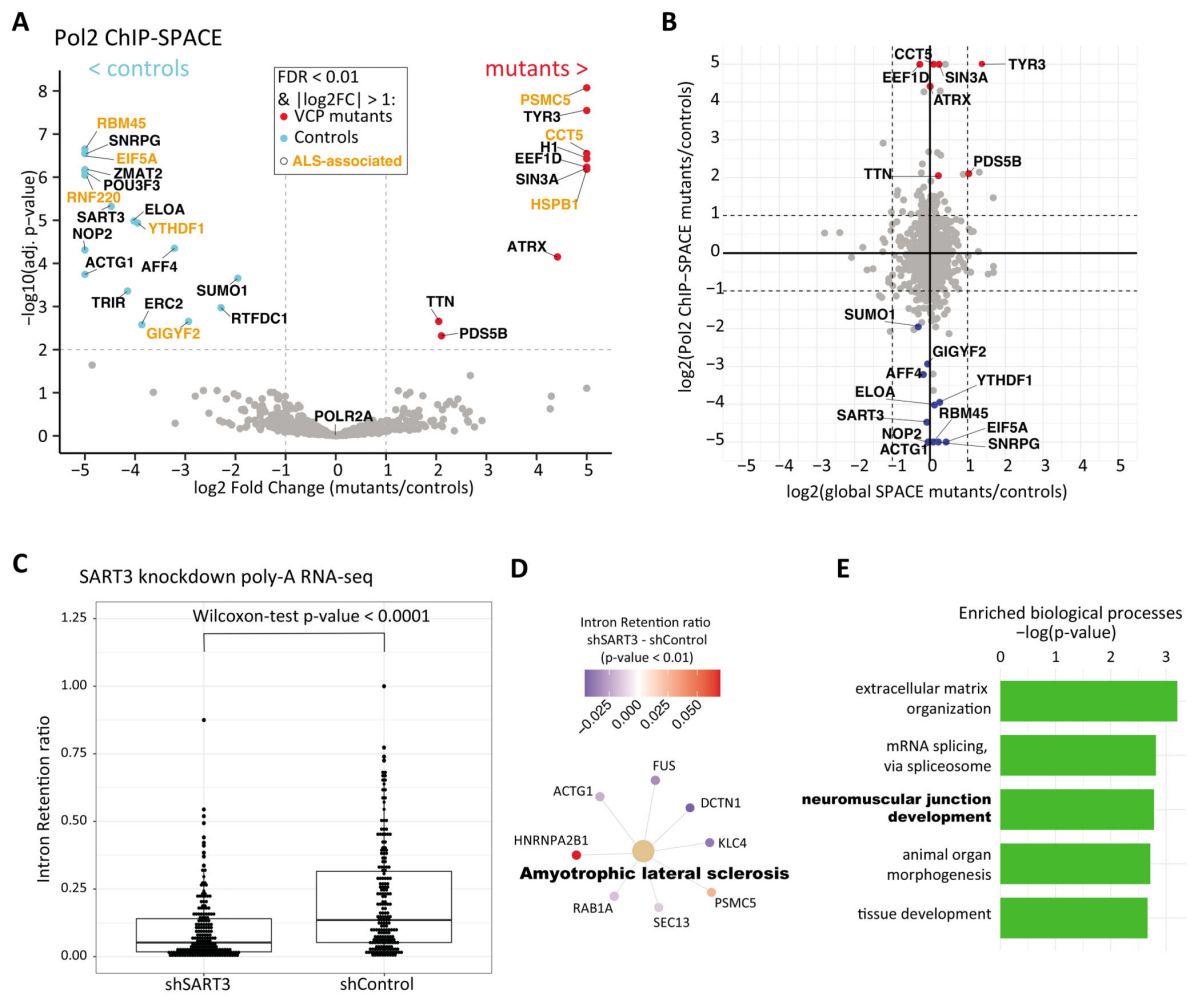
Statistically significant proteins in RNAP2 complex of VCP mutant and control cells. (A) The volcano plot shows the proteins that differentially bind RNAP2 in VCP mutant and control cells with red and blue dots, respectively. The ALS-associated proteins are indicated by the orange color. (B) Global quantification of chromatin-binding proteins and comparing with the RNAP2 ChIP-SPACE fold changes. (C) SART3 was knockdown in HEP G2 cells, and the effect on the transcriptome was assessed using poly-A RNA-seq. Using IRfinder we calculated Intron Retention (IR) ratios for each transcript. The mean of IR ratios between SART3 knockdown and control was compared using a paired Wilcoxon-test. (D) KEGG pathway gene set enrichment analysis among the genes with IRfinder p-value < 0.01. Node colors show the difference of IR ratios (shSART3 – shControl). (E) GO enrichment analysis using genes with IRfinder p-value < 0.01. The rest of the genes were used as the background.

Using Bayesian-moderated t-test and Benjamini-Hochberg procedure to control false detection rate (FDR), we shortlisted the proteins that were statistically significant between the mutant and control groups. Of 1126 RNAP2-associated proteins identified (Supplementary Table S1), 18 were significantly enriched in the VCP mutant cells, and 10 were significantly depleted relative to the WT controls (FDR < 0.01 and log2-fold-change > 1, Figure 3A). POLR2A showed log2-fold-change = 0.02, which indicates that immunoprecipitation efficiency was almost equal among the samples. We found important players in neurodegenerative disorders that were highly depleted in the VCP mutant group: RBM45, EIF5A, RNF220, YTHDF1, and GIGYF2. For instance, nuclear and cytoplasmic RBM45 inclusions were reported in neurons and glia of ALS disease, TDP-43 associated frontotemporal lobar degeneration, and Alzheimer’s disease ^19^. RBM45 is involved in RNA splicing and spliceosome functions, however, the precise function is not clearly understood. Several previous studies have shown that mutant RBM45 relocates to the cytoplasm and co-aggregates with other RBPs such as TDP-43 into toxic immunoreactive inclusions ^20–22^.

EIF5A is a U6 snRNA-binding protein that plays important roles in neurodevelopment. Interestingly, a lysine residue (K50) in EIF5A is post-translationally modified to hypusine which regulates TDP-43 accumulation and aggregation ^23^. So far EIF5A is the only identified protein in the cells with hypusine modification which indicates the importance of this protein in human neurodevelopment ^24^.

Besides, RNF220 is an E3-ubiquitin ligase that was highly depleted in mutant cells. Notably, RNF220 poly-ubiquitinates TDP-43 and targets it for proteasomal degradation. It has been reported that RNF220 heterozygote mutant mice develop ALS pathology ^25^. GIGYF2 is an RBP, and heterozygous GIGYF2^+/-^ mice develop adult-onset neurodegeneration ^26^. YTHDF1 is a reader of m^6^A-modified mRNAs and is required for axon regeneration in neurons ^27^.

Furthermore, AFF4 and ELOA, critical factors in the RNAP2 transcription elongation ^28, 29^, also showed significant depletion from RNAP2 complexes in VCP mutant cells. Thus, our data suggest a possible RNAP2 elongation defect in mutant cells.

Conversely, we observed three RBPs that are more abundantly co-localized with RNAP2 in mutant cells: CCT5, EEF1D, and HSPB1. Remarkably, HSBP1 is a chaperone that preserves the proteins in a folding-competent state, and mutations of HSPB1 affect motor neurons of the peripheral nervous system ^30^. CCT5 is also a chaperonin-containing T-complex (TRiC) component, which controls protein aggregation. In Alzheimer’s disease, the expression of CCT5 is decreased, and mutation of CCT5 leads to sensory neuropathy ^31^. Interestingly, EEF1D is not only a translation elongation factor, but the long isoform is also a transcription factor that induces heat-shock-responsive genes ^32^. In summary, chaperones and proteins involved in stress response are co-localized more abundantly with RNAP2 in the VCP-mutant cells.

To understand if the identified proteins are globally changed on chromatin or specifically on the RNAP2 complex, we performed global SPACE to compare the total amount of chromatin-binding proteins between mutant and wild-type cells. None of the proteins with perturbed co-localization with RNAP2 showed any significant changes in their global chromatin-binding (Figure 3B). For example, RBM45 copurifies with RNAP2 32-fold higher from the control cells than the mutant cells but doesn’t show a significant change in chromatin using global SPACE (fold change = 1.1). Thus, the changes observed in RNAP2-associated factors do not result from global changes in the proteome of chromatin.

Altogether, our data indicate that several pathways including proteostasis and RNA splicing are perturbed among the RNAP2-associated proteins in the mutant cells. Some of these factors were already studied individually in the context of ALS disease; thus, underscores the validity of our results. We next sought to verify SART3 as one of the uncharacterized proteins in the context of ALS disease.

### SART3 knockdown perturbs splicing of neuromuscular junction development genes

SART3 functions in recycling the splicing factors by promoting the reassembly of U4/U6 snRNP after splicing ^33^. SART3 directly interacts with SNRPG which is a core component of the spliceosomal U1, U2, U4 and U5 snRNPs. Downregulation of SART3 and a few other splicing factors in the RNAP2 complex suggests splicing is perturbed in the mutant cells. To validate this hypothesis, we searched the publicly available datasets and found SART3 knockdown combined with poly-A RNA-seq data ^34^. In fact, aberrant alternative splicing such as intron retention (IR) is frequently observed in *post-mortem* samples of ALS patients ^35, 36^. We, therefore, compared the SART3 knockdown with the control to quantify intron retention events using IRfinder ^37^ a package developed for detecting IR in mRNA-seq data. In general, we observed lower IR in the SART3 knockdown cells (Figure 3C and Supplementary Table 2). We then analyzed the genes with significantly differential IR events between the knockdown and control (p-value < 0.01) using KEGG pathway gene set enrichment. Surprisingly, the only enriched pathway that we detected was ALS (Figure 3D). Gene Ontology (GO) analysis of the genes with significantly differential IR events indicates that neuromuscular junction development is among the top 3 overrepresented biological processes (Figure 3E). Indeed, previous studies have shown that synaptic connections between motor neurons and skeletal muscles are highly disturbed in ALS such that neuromuscular junction damages may occur in the initial stages of the disease ^38^. Furthermore, enrichment of extracellular matrix organization genes such as different collagens points toward other aspects of the VCP mutations that are associated with musculoskeletal disorders ^39^. Collectively, this result strongly links SART3 deficiency to ALS-associated genes via IR events and independently verifies our previous observation that SART3 is downregulated in the RNAP2 complex of the VCP mutant cells.

## Discussion

**Figure 4:**
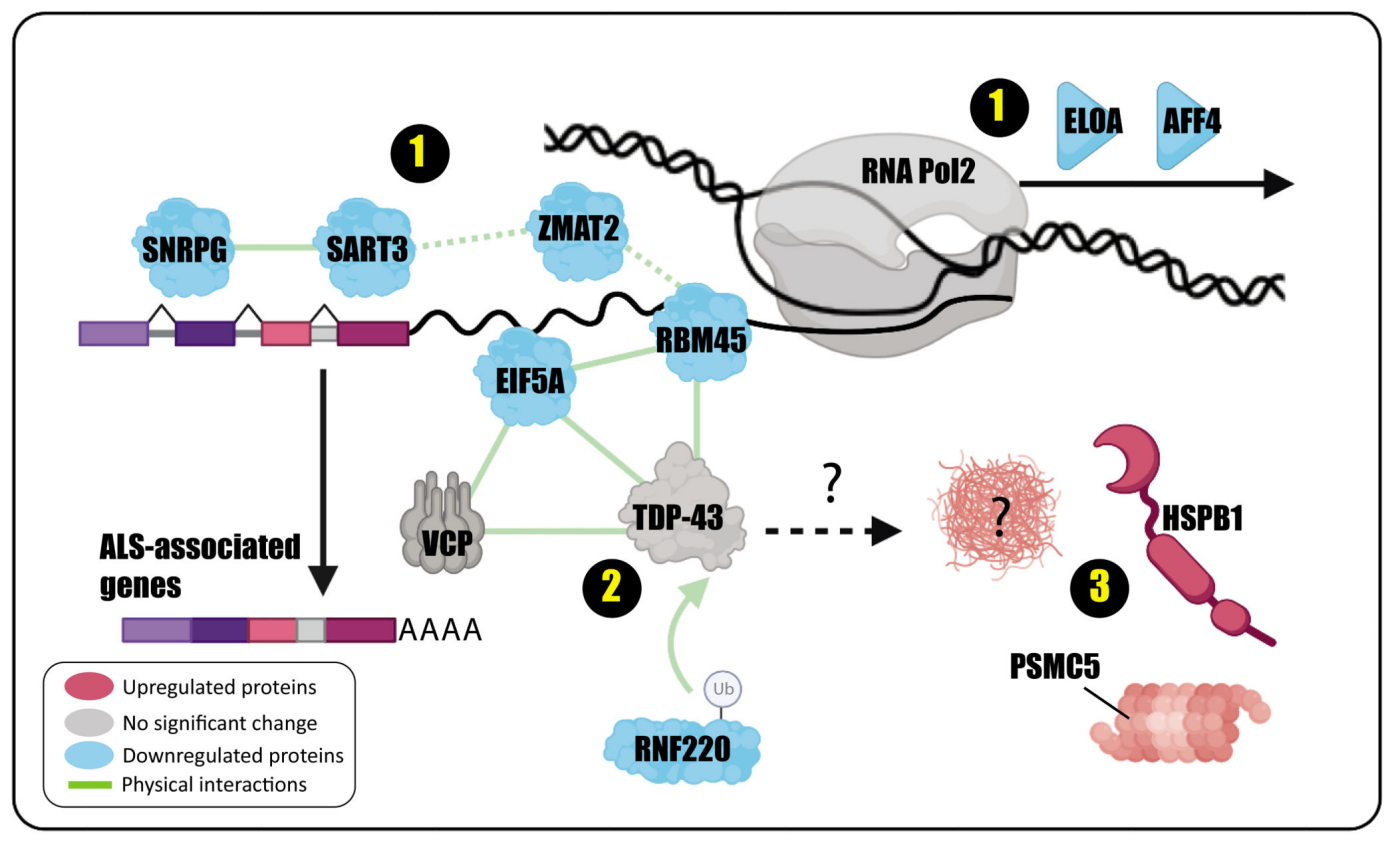
Three perturbed pathways in the transcriptional machinery of the VCP mutant cells. 1- Splicing and transcription machinery: Several proteins involved in mRNA splicing show a significant reduction in the mutant cells. Additionally, lower ELOA and AFF4 affect RNAP2 elongation which also affect alternative splicing. At least, depletion of SART3 impairs intron retention in ALS-associated genes. 2- TDP-43 regulation: RNF220, RBM45 and EIF5A that are regulators of TDP-43 show diminished binding to RNAP2. RNF220 is an E3-ubiquitin ligase that targets TDP-43 for proteasomal degradation. The lack of RNF220 in the RNAP2 complex of the mutant cells potentially makes TDP-43 susceptible to accumulation and aggregation close to the transcription machinery. 3- Protein re-folding and degradation: The mutant cells recruit heat shock proteins, such as HSPB1 and PSMC5 (a regulating subunit of proteasome) as a defensive mechanism to alleviate protein aggregation.

VCP mediates ubiquitin-dependent protein extraction from chromatin through the ubiquitin-proteasome system ^2^. Inactivation of VCP leads to protein-induced chromatin stress and aggregation, which has been studied in the context of DNA repair and replication ^2^. Our data provide a model to understand how VCP mutations affect transcriptional machinery in an ALS model system. Our findings reveal at least 3 pathways that are perturbed in the mutant cells (Figure 4). The first pathway is related to transcription and splicing machinery which are highly interconnected. Our results indicate decreased binding of RNAP2 elongation factors AFF4 and ELOA in the mutant cells. AFF4 is a core component of the super elongation complex and acts as a scaffold protein for other proteins that together are required for increasing the catalytic rate of RNA RNAP2 ^35^. ELOA (Elongin A) is the largest subunit of the elongin complex, which stimulates RNA RNAP2 elongation ^40^. ELOA regulates the transition of paused RNAP2 to the elongation state ^41^. While an optimal rate of transcription elongation is needed for proper pre-mRNA splicing ^42^, combined deficiency of AFF4 and ELOA might indicate inconsistent RNAP2 elongation in the mutant cells which leads to aberrant splicing. Furthermore, several RBPs involved in mRNA processing and splicing such as ZMAT2, RBM45, SNRPG, and SART3 are decreased in the proximity of RNA RNAP2 in the VCP mutant cells. Interestingly, knockdown of SART3 leads to perturbation of IR in ALS-associated genes. Although this experiment was carried out in HEP G2 cells, ALS pathway and neuromuscular junction development genes show significant enrichment. Noteworthy, some genes such as FUS show lower IR in SART3 knockdown in comparison to the control, whereas HNRNPA2B1 have higher IR. In general, transcript isoforms with IR trigger non-sense mediated decay which leads to downregulation of the proteins ^43^. Thus, perturbation of IR in ALS-associated genes probably deregulates their balance of translation and proteostasis.

Secondly, we found RBM45, EIF5A, and RNF220 as direct interactors and regulators of TDP-43 that are dramatically reduced in the mutant cells. TDP-43 aggregation is observed in many neurodegenerative disorders ^44^, including > 97% of sporadic and familial ALS patients ^45^. However, TDP-43 is mutated in 1%-4% of the cases ^46^. This indicates that regulators of TDP-43 are affected in ALS disease, thus allowing TDP-43 to accumulate and aggregate. Recently, RNF220 was introduced as a regulator of TDP-43 that targets it for proteasome degradation by polyubiquitination ^25^. RNF220 is required for the function and development of the cerebellum and central motor neurons in mice ^47^. Interestingly, RNF220+/- mice accumulate TDP-43 in their cytoplasm. The mice also develop phenotypes similar to ALS such as hindlimb paralysis, muscle wasting, and neuronal loss ^25^. We speculate that reduced RNF220 in RNAP2 complexes of VCP mutant cells could be an early step that leads to TDP-43 aggregation.

The third pathway is upregulated in the mutant cells, as heat shock proteins such as HSPB1 and CCT5 show increased binding to the RNAP2 complex in the mutant cells. Mutations of HSPB1have been reported in Charcot-Marie-Tooth neuropathy, distal hereditary motor neuropathies, and ALS ^48^. We also observed increased binding of PSMC5, a subunit of 19s proteasome which has ATPase activity to unfold the ubiquitinated proteins and has chaperone-like activity ^4^. The increased binding of heat shock proteins and chaperones provides a protective mechanism against protein aggregation and misfolding.

Taken together, our study sheds light on the disrupted pathways in the transcriptional machinery in an ALS model system with VCP mutations. This situation could potentially lead to proteinopathy and aberrant alternative splicing, which is observed in neurodegeneration. In the future, RNAP2 ChIP-SPACE would be a valuable approach for further analyses of additional cell types and samples from ALS patients with mutations in multiple genes or sporadic ALS cases to gain a more comprehensive insight into the pathological mechanism of the disease.

## Methods

### Cell culture and neural differentiation

The hiPS cells were cultured on Geltrex (Life Technologies), grown using Essential 8 Medium (Life Technologies), passaged using EDTA (Life Technologies, 0.5 mM), and maintained at 37°C incubators with 5% CO_2_. The hiPS cells were differentiated toward motor neuron precursors using a previously described protocol ^13^.

### Global and ChIP-SPACE

SPACE experiments were carried out essentially as described previously ^49^. The cells were fixed in their medium by adding formaldehyde (final concentration 1%). Then the cells were collected by cell lifters and washed with PBS. Three mutant and three control cell lines were compared using Pol2 ChIP-SPACE. For each cell line, 2 independent replicates were prepared. The cells were resuspended in TE buffer with Tritone 1% for 5min on ice. The cells were washed with LB3 buffer (Tris.HCl 10 mM pH 8, NaCl 100 mM, EDTA 1 mM, EGTA 0.5 mM, Na-deoxycholate 0.1%, and N-lauroylsarcosine 0.5%). Chromatin was sheared by Bioruptor Pico for 10 cycles: 30s on and 30s off. The sheared chromatin was spun at 12000g for 10min to remove the cell debris. To the samples, Tritone X100 was added (final concentration 1%). Then 10ug pan-RNAP2 antibody (Bethyl Laboratories, A304-405A) or normal IgG (Cell Signalling Tech, 2729S) were added, and the samples were rotated overnight in the cold room. The day after, the samples were again spun at 12000g for 10min. The supernatant was transferred to new tubes, and 40ul Dynabeads magnetic protein A was added to the samples. After 2-3 hours rotating in cold room, the samples were washed 5 times with IP buffer (Tris-HCl 50mM pH 7.5, Triton X-100 1%, NP-40 0.5%, EDTA 5mM). Finally, the beads were resuspended in 100ul IP buffer, and 10ul RNase A (10mg/ml) was added to the samples. The samples were incubated at 37 °C with 500 RPM agitation. After that, 500ul SPACE lysis buffer (Guanidinium thiocyanate 4M, Tris HCl 100mM, Sarkosyl 2%, EDTA 10mM) was added to the samples. After vigorous vortexing, 400ul 2-propanol was added. Then 30ul DNA-binding beads (Thermo Scientific 4489112) were added to the samples and vortexed vigorously. After 10min, the beads were separated on the magnet and washed with wash buffers, as mentioned in the original protocol.

For global SPACE, three mutant and three control cell lines were compared. For each cell line, 3 independent replicates were prepared. After fixation and lifting the cells, the cells were directly dissolved in 1ml lysis buffer (Guanidinium thiocyanate 4M, Tris HCl 100mM, Sarkosyl 2%, EDTA 10mM). After vortexing, 0.8ml 2-propanol was added. Then 50ul DNA-binding beads were added, and the samples were vortexed. After 10min, the beads were isolated on the magnet and washed with wash buffers, as mentioned in the original protocol. The beads were resuspended in Tris HCl pH 8 10mM by 3 cycles of sonication. 10ul RNase A (10mg/ml) was added to the samples. The samples were incubated at 37 °C with 500 RPM agitation. Once again, lysis buffer and 2-propanol were added to the beads, and they were washed using wash buffers.

### Mass spectrometry

After washing the DNA-binding beads, the beads were resuspended in 25ul Ambic 100mM plus dithiothreitol (DTT) 10mM final concentration. Then the samples were incubated at 95°C for 10min to reduce the disulfide bonds and reverse the crosslinking. The cysteines were then alkylated with Iodoacetamide (40mM) for 15min in the dark. Iodoacetamide was neutralized by adding DTT (10mM) again. The proteins were digested with 300 ng Trypsin/LysC-mix (Promega, V5071) for 14-16 hours at 37 °C. Finally, the peptides were desalted using Ziptips with 0.6 μL C_18_resin (Merck). After the sample preparation, peptides were separated on a 50 cm, 75 μm I.D. Pepmap column during 60 min runs for ChIP-SPACE, or 120 min runs for global SPACE samples. The mass spectrometer (Orbitrap Fusion Lumos) worked with a universal data-dependent acquisition Thermo Scientific HCD-IT method while injecting the peptides. The mass spectrometer was controlled by Xcalibur 4.2 and Tune 3.1.

### Mass spectrometry and transcriptome data analysis

Mass spectrometry RAW data were analyzed using MaxQuant (2.0.1.0)^50^. The spectra were searched against the UniProt (Swissprot) (Homo sapiens) and contaminants databases. Trypsin/P and LysC were chosen as enzyme specificity, and a maximum of two missed cleavages were allowed. Cysteine carbamidomethylation was selected as the fixed modification, and methionine oxidation and protein N-terminal acetylation were variable modifications. The global false discovery rate for both protein and peptides was set to 1%. The match-from-and-to, re-quantify, and intensity-based absolute quantification (iBAQ) options were enabled. The other parameters were set on default. After the MS data analysis by MQ, the protein groups were processed in RStudio using R version 4.0.0. The proteins only identified by site, Reverse, and potential contaminants as well as proteins identified using normal IgG control were filtered out. The iBAQ values were log2 transformed and normalized using PreProcess package. If iBAQ values of a protein were missing in all six control samples and it was not missing in at least 3 VCP mutant samples, the missing values in the control samples were imputed with the minimum iBAQ value of the control samples. The same imputation conditions were applied for the VCP mutant samples if all six samples had no values and at least three control samples were quantified. The limma package ^51^ was used to determine Bayesian moderated t-test p-values and Benjamini-Hochberg (BH) adjusted p-values (adj. p-value or FDRs). We considered log2-fold-change > 1 and and adj. p-value <0.01 as significantly enriched proteins. KEGG pathway enrichment and gene set enrichment analysis was performed by enrichplot and clusterProfiler ^52^ packages in R. Gene Ontology (GO) and other information were downloaded from DAVID Gene Ontology database.

SART3 knockdown RNA-seq data were obtained from Nostrand et. al. ^34^. We compared the knockdown and control using IRfinder ^37^ which calculates IR ratio for each transcript as follows: # Intronic reads / (# Intronic reads + # flanking exonic reads). We applied IRFinder default parameters to determine differentially retained introns and to remove the IRs with the following warnings: LowCover, LowSplicing, and MinorIsoform. We considered differential p-values < 0.01 as statistically significant.

## Supplementary Material

Supplementary material

Supplementary Table 1

Supplementary Table 2

## Data Availability

The proteomics data were deposited to jPOST database. The accession numbers are PXD034483 for ProteomeXchange and JPST001663 for jPOST. URL: https://repository.jpostdb.org/preview/116927318562a51ada6c308 Access key 2790
