# RNA polymerase II-associated proteins reveal pathways affected in VCP-related amyotrophic lateral sclerosis

**DOI:** 10.1093/brain/awad046

**Published:** 2023-02-15

**Authors:** Mahmoud-Reza Rafiee, Sara Rohban, Karen Davey, Jernej Ule, Nicholas M Luscombe

**Affiliations:** The Francis Crick Institute, London NW1 1AT, UK; The Francis Crick Institute, London NW1 1AT, UK; The Francis Crick Institute, London NW1 1AT, UK; The Francis Crick Institute, London NW1 1AT, UK; Department of Neuromuscular Diseases, UCL Queen Square Institute of Neurology, London, WC1N 3BG, UK; UK Dementia Research Institute Centre, King’s College London, London, SE5 9RX, UK; The Francis Crick Institute, London NW1 1AT, UK; UCL Genetics Institute, University College London, London, WC1E 6BT, UK; Genomics and Regulatory Systems Unit, Okinawa Institute of Science & Technology Graduate University, Okinawa 904-0495, Japan

**Keywords:** RNA polymerase II, SPACE, proteomics, ALS, VCP

## Abstract

Valosin-containing protein (VCP) is a hexameric ATPase associated with diverse cellular activities. Genetic mutations in VCP are associated with several forms of muscular and neuronal degeneration, including amyotrophic lateral sclerosis (ALS). Moreover, VCP mediates UV-induced proteolysis of RNA polymerase II (RNAPII), but little is known about the effects of VCP mutations on the transcriptional machinery. Here, we used silica particle-assisted chromatin enrichment and mass spectrometry to study proteins co-localized with RNAPII in precursor neurons differentiated from VCP-mutant or control induced pluripotent stem cells. Remarkably, we observed diminished RNAPII binding of proteins involved in transcription elongation and mRNA splicing in mutant cells. One of these is SART3, a recycling factor of the splicing machinery, whose knockdown leads to perturbed intron retention in several ALS-associated genes. Additional reduced proteins are RBM45, EIF5A and RNF220, mutations in which are associated with various neurodegenerative disorders and are linked to TDP-43 aggregation. Conversely, we observed increased RNAPII binding of heat shock proteins such as HSPB1. Together, these findings shed light on how transcription and splicing machinery are impaired by VCP mutations, which might contribute to aberrant alternative splicing and proteinopathy in neurodegeneration.

## Teaser

Dysregulation of proteostasis and RNA metabolism are increasingly recognised as critical drivers of neurodegenerative disease. Rafiee *et al*. focus on RNA polymerase II as the converging point of these two processes, and examine how transcriptional machinery and alternative splicing are affected by ALS-associated VCP mutations.

## Introduction

Valosin-containing protein (VCP; p97 or Cdc48) is a highly conserved member of type II AAA+ (ATPase associated with diverse cellular activities) superfamily of proteins and is a central component of the ubiquitin protease system that is integral to cellular proteostasis.^1^ VCP plays crucial roles in multiple cellular processes, including protein degradation, intracellular trafficking, DNA repair and replication, transcription and cell-cycle regulation.^2^ On chromatin, VCP was shown to facilitate ubiquitin-driven segregation of several proteins from DNA,^3^ including proteins involved in the termination of replication.^4^ VCP directly interacts with RNA polymerase II (RNAPII) and plays an important role in mediating RNAPII degradation during DNA damage^5,6^ or unstable elongation.^7^ However, little is known about how mutations of VCP affect RNAPII and the associated factors in neurodegenerative diseases.

In fact, VCP mutations were reported in a wide range of disorders, including frontotemporal dementia (FTD), Charcot–Marie–Tooth disease, inclusion body myopathy, Paget disease of bone, Parkinson’s disease and amyotrophic lateral sclerosis (ALS).^8^ While cytoplasmic accumulation and nuclear loss of TDP-43 in motor neurons are considered a hallmark of the ALS disease at late stages,^9^ abnormal alternative splicing is an earlier sign of the disease,^10^ which implies RNAPII accessory proteins are probably involved in the onset of the disease. Previous studies have shown that alternative splicing has a crucial role in brain development,^11^ ageing^12^ and neurodegenerative disorders^13^; however, the link between VCP mutations, RNAPII accessory factors, aberrant alternative splicing and TDP-43 proteinopathy is not clear.

Here, we studied RNAPII-associated proteins in patient-derived human induced pluripotent stem (iPS) cells with VCP mutations undergoing neurogenesis. The patients contained either the R155C (most prevalent *VCP* mutation) or R191Q mutations, which are associated with both ALS and FTD.^8^ Using chromatin immunoprecipitation combined with silica particle-assisted chromatin enrichment mass spectrometry (SPACE-MS), we compared mutant and control neural precursors to identify deregulated proteins. The advantages of the SPACE method in comparison to a conventional ChIP-MS method are as follows^14^: (i) removing artificial protein–protein interactions by applying a double purification strategy and stringent washing steps; (ii) improving sensitivity of the experiment by on-bead digestion of the proteins which is more efficient than in-gel or in-solution digestion; (iii) removing detergents and other harmful reagents for MS; and (iv) removing artificial interactions allows for more accurate detection and quantification of genuine interactions. Our results indicate that in the mutant cells several mRNA splicing factors including regulators of TDP-43 are depleted, while chaperones are upregulated in association with RNAPII. Our approach thus provides an insight into the perturbed pathways in ALS.

## Materials and methods

### Ethics statement

All patients and healthy control subjects in this study were informed, and consent was obtained. The experiments were performed in compliance with the approved regulations and guidelines by UCLH’s National Hospital for Neurology and Neurosurgery and UCL’s Institute of Neurology joint research ethics committee (09/0272).

### Cell culture and neural differentiation

The human iPS (hiPS) cell lines were generated and validated by previous studies.^15,16^ The patients contained either the R155C (most prevalent *VCP* mutation) or R191Q mutations, and both displayed autosomal dominant inclusion body myopathy and FTD,^16^ and the same mutations are often also linked to ALS.^8^ To generate hiPS cells, episomal reprogramming plasmids were transfected into the fibroblast cells.^17^ Details of the cell lines and patients are provided in Supplementary Table 1. spreadsheet 3. The hiPS cells were cultured on Geltrex (Life Technologies), grown using Essential 8 Medium (Life Technologies), passaged using EDTA (Life Technologies, 0.5 mM) and maintained at 37°C incubators with 5% CO_2_. The hiPS cells were differentiated towards motor neuron precursors using an adapted version of a previously published protocol.^15,18^ Briefly, iPSs were differentiated to neuroepithelium in a chemically defined medium consisting of DMEM/F12 GlutaMAX, Neurobasal, L-glutamine, N2 supplement, non-essential amino acids, B27 supplement, β-mercaptoethanol (all from Life Technologies) and insulin (Sigma). The following small molecules were added to the medium from Days 0 to 7: 1 μM dorsomorphin (Millipore), 2 μM SB431542 (Tocris Bioscience) and 3 μM CHIR99021 (Miltenyi Biotec). At Day 8, the neuroepithelial layer was treated with dispase (GIBCO, 1 mg/ml), and seeded onto laminin-coated plates and then patterned for 7 days with 0.5 μM retinoic acid and 1 μM purmorphamine (Sigma).

### Global/ChIP-SPACE and mass spectrometry

SPACE experiments were carried out essentially as described previously.^14^ The cells were fixed in their medium by adding formaldehyde (final concentration 1%), collected using cell lifters and washed with PBS. Three mutant and three control cell lines were compared using RNAPII ChIP-SPACE. For each cell line, two independent replicates were prepared. The cells were resuspended in TE buffer with Triton 1% for 5 min on ice. The cells were washed with LB3 buffer (Tris-HCl 10 mM pH 8, NaCl 100 mM, EDTA 1 mM, EGTA 0.5 mM, Na-deoxycholate 0.1%, and *N*-lauroylsarcosine 0.5%). Chromatin was sheared using a Bioruptor Pico for 10 cycles: 30 s on and 30 s off. The sheared chromatin was spun at 12 000*g* for 10 min to remove cell debris. To the samples, Triton X-100 was added (final concentration 1%). Then 10 μg pan-RNAPII antibody (Bethyl Laboratories, A304–405A) or normal IgG (Cell Signalling Tech, 2729S) were added, and the samples were rotated overnight in the cold room. The next day, the samples were again spun at 12 000*g* for 10 min. The supernatant was transferred to new tubes, and 40 μl Dynabeads magnetic protein A was added to the samples. After 2–3 h rotating in cold room, the samples were washed five times with IP buffer (Tris-HCl 50 mM pH 7.5, Triton X-100 1%, NP-40 0.5%, EDTA 5 mM). Finally, the beads were resuspended in 100 μl IP buffer and 10 μl RNase A (10 mg/ml) was added to the samples. The samples were incubated at 37°C with 500 rpm agitation, then 500 μl SPACE lysis buffer (guanidinium thiocyanate 4 M, Tris-HCl 100 mM, Sarkosyl 2%, EDTA 10 mM) was added to the samples. After vigorous vortexing, 400 μl 2-propanol was added. Then 30 μl DNA-binding beads (Thermo Scientific 4489112) were added to the samples and vortexed vigorously. After 10 min, the beads were separated on the magnet and washed with wash buffers, as mentioned in the original protocol.

For global SPACE, three mutant and three control cell lines were compared. For each cell line, three independent replicates were prepared. After fixation and lifting the cells, the cells were directly dissolved in 1 ml lysis buffer (guanidinium thiocyanate 4 M, Tris-HCl 100 mM, Sarkosyl 2%, EDTA 10 mM). After vortexing, 0.8 ml 2-propanol was added. Then 50 μl DNA-binding beads were added and the samples were vortexed. After 10 min, the beads were isolated on the magnet and washed with wash buffers, as mentioned in the original protocol. The beads were resuspended in Tris-HCl pH 8 10 mM by three cycles of sonication. Ten microlitres of RNase A (10 mg/ml) was added to the samples. The samples were incubated at 37°C with 500 RPM agitation. Once again, lysis buffer and 2-propanol were added to the beads, and they were washed using wash buffers. After washing the DNA-binding beads, the beads were resuspended in 25 μl Ambic 100 mM plus dithiothreitol (DTT) 10 mM final concentration. Then the samples were incubated at 95°C for 10 min to reduce the disulphide bonds and reverse the crosslinking. The cysteines were then alkylated with iodoacetamide (40 mM) for 15 min in the dark. Iodoacetamide was neutralized by adding DTT (10 mM) again. The proteins were digested with 300 ng trypsin/LysC-mix (Promega, V5071) for 14–16 h at 37°C. Finally, the peptides were desalted using Ziptips with 0.6 µl C_18_ resin (Merck). After the sample preparation, peptides were separated on a 50 cm, 75 µm I.D. Pepmap column during 60 min runs for ChIP-SPACE, or 120 min runs for global SPACE samples. The mass spectrometer (Orbitrap Fusion Lumos) worked with a universal data-dependent acquisition Thermo Scientific HCD-IT method while injecting the peptides. The mass spectrometer was controlled by Xcalibur 4.2 and Tune 3.1.

### Mass spectrometry data analysis

Mass spectrometry RAW data were analysed using MaxQuant (2.0.1.0).^19^ The spectra were searched against the UniProt (Swissprot) (*Homo sapiens*) and contaminants databases. Trypsin/P and LysC were chosen as enzyme specificity, and a maximum of two missed cleavages were allowed. Cysteine carbamidomethylation was selected as the fixed modification, and methionine oxidation and protein N-terminal acetylation were variable modifications. The global false discovery rate for both protein and peptides was set to 1%. The match-from-and-to, re-quantify, and intensity-based absolute quantification (iBAQ) options were enabled. The other parameters were set on default. After the MS data analysis by MaxQuant, the protein groups were processed in RStudio using R version 4.0.0. The proteins only identified by site, Reverse, and potential contaminants as well as proteins identified using normal IgG control were filtered out. The iBAQ values were log2 transformed and normalized using PreProcess package. If iBAQ values of a protein were missing in all six control samples and it was not missing in at least three VCP mutant samples, the missing values in the control samples were imputed with the minimum iBAQ value of the control samples. The same imputation conditions were applied for the VCP mutant samples if all six samples had no values and at least three control samples were quantified. The limma package^20^ was used to determine Bayesian moderated *t*-test *P*-values and Benjamini–Hochberg adjusted *P*-values (adj. *P*-value or FDRs). We considered log2-fold-change > 1 and and adj. *P*-value <0.01 as significantly enriched proteins. Kyoto Encyclopedia of Genes and Genomes (KEGG) pathway enrichment and gene set enrichment analysis was performed by enrichplot and clusterProfiler^21^ packages in R. Gene Ontology (GO) and other information were downloaded from DAVID Gene Ontology database.

### SART3 knockdown RNA-seq and RT-qPCR

SART3 knockdown and RNA-seq data (Fig. 3C–E) were obtained from Van Nostrand *et al*.^22^ We compared the knockdown and control using IRfinder,^23^ which calculates intron retention ratio for each transcript as follows: number of intronic reads (number of intronic reads + number of flanking exonic reads). We applied IRFinder default parameters to determine differentially retained introns and to remove the intronic reads with the following warnings: LowCover, LowSplicing and MinorIsoform. We considered differential *P*-values < 0.01 as statistically significant.

To validate the role of SART3 in intron retention of *FUS* and *HNRNPA2B1*, we knocked down SART3 in BJ cells (ATCC CRL-2522) using three different siRNAs separately (Silencer Select, Life Technologies s18770, s18771, s18772) and siControl (Silencer Select negative control 2, Life Technologies 4390846) as the negative control. The cells were transfected using Lipofectamine RNAiMAX (Thermofisher) using three independent replicates (*n* = 3) for each siRNA. About 48 h after the transfection, the cells were harvested using the lysis buffer of the RNeasy kit (Qiagen). Purified RNA (∼500 ng) was subjected to Turbo DNase treatment (Thermofisher) using 4 U of the enzyme for 30 min at 37°C. cDNA was synthesized by PrimeScript RT reagent kit (Takara) using only Oligo-dT to target mature mRNAs for qPCR. We used noRT control as a negative control to verify that genomic DNA was removed. We used the IRfinder report (Supplementary Table 2) to design primers for the intronic regions of *FUS* (intron 7) and *HNRNPA2B1* (intron 11). The specificity of the PCR products was checked by primer blast and by melting temperature (Tm) of the PCR products. To measure intron retention, we normalized the expression of intronic regions with the exons of the same gene and with the *GAPDH* expression. The results were compared with siControl to assess the effect of *SART3* knockdown on the intron retention of *FUS* and *HNRNPA2B1* as examples of ALS-associated genes whose intron retentions were determined to be perturbed by IRfinder using the public data. The siSART3s and the siControl were compared using a one-way *t*-test to determine significant perturbations. For the primer sequences, refer to Supplementary Table 2. Finally, the ratio of intron retention was calculated in siSART3 over the siControl.

### Data availability

The proteomics data were deposited to jPOST database. The accession numbers are PXD034483 for ProteomeXchange and JPST001663 for jPOST.

## Results

### Pathways involved in neurodegenerative disorders are enriched by RNAPII-associated factors

**Figure 1:**
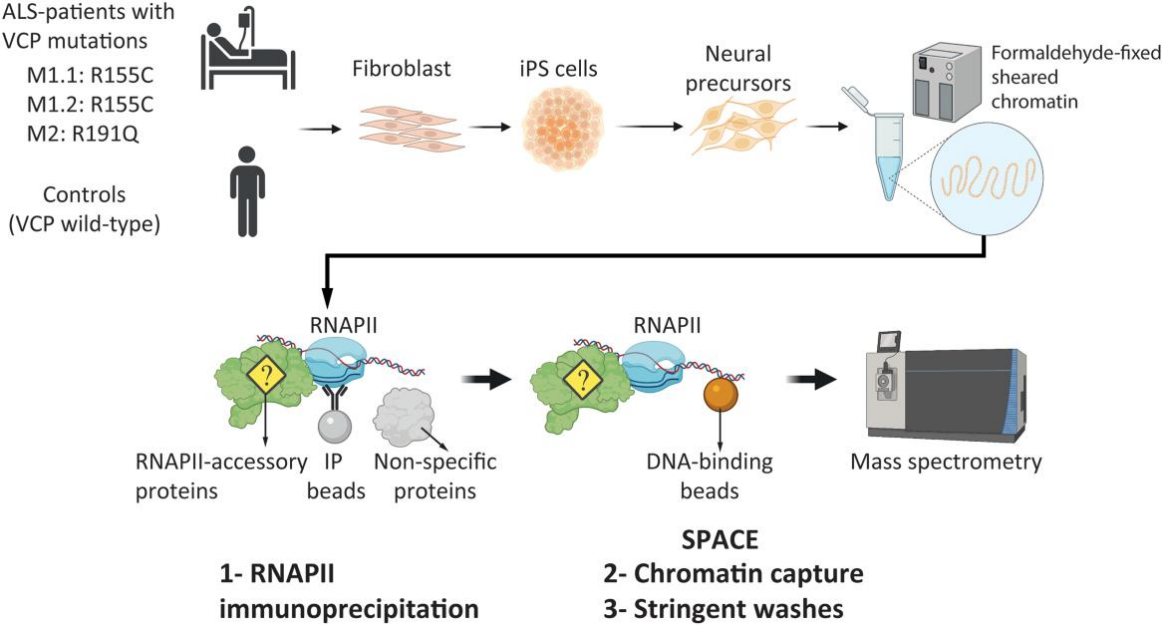
**Schematic presentation of the experiments**. Three ALS patient iPS cell lines and three control iPS cell lines were differentiated to neural precursors until Day 14. For each cell line, two replicates were used. The cells were fixed by formaldehyde and subjected to the ChIP procedure to fragment chromatin using sonication. After the immunoprecipitation by a pan-RNAPII antibody, chromatin fragments were purified using silica magnetic DNA-binding beads (the SPACE procedure) to remove artificial interactions and to identify co-localized proteins with RNAPII. The figure was created with BioRender.com.

**Figure 2:**
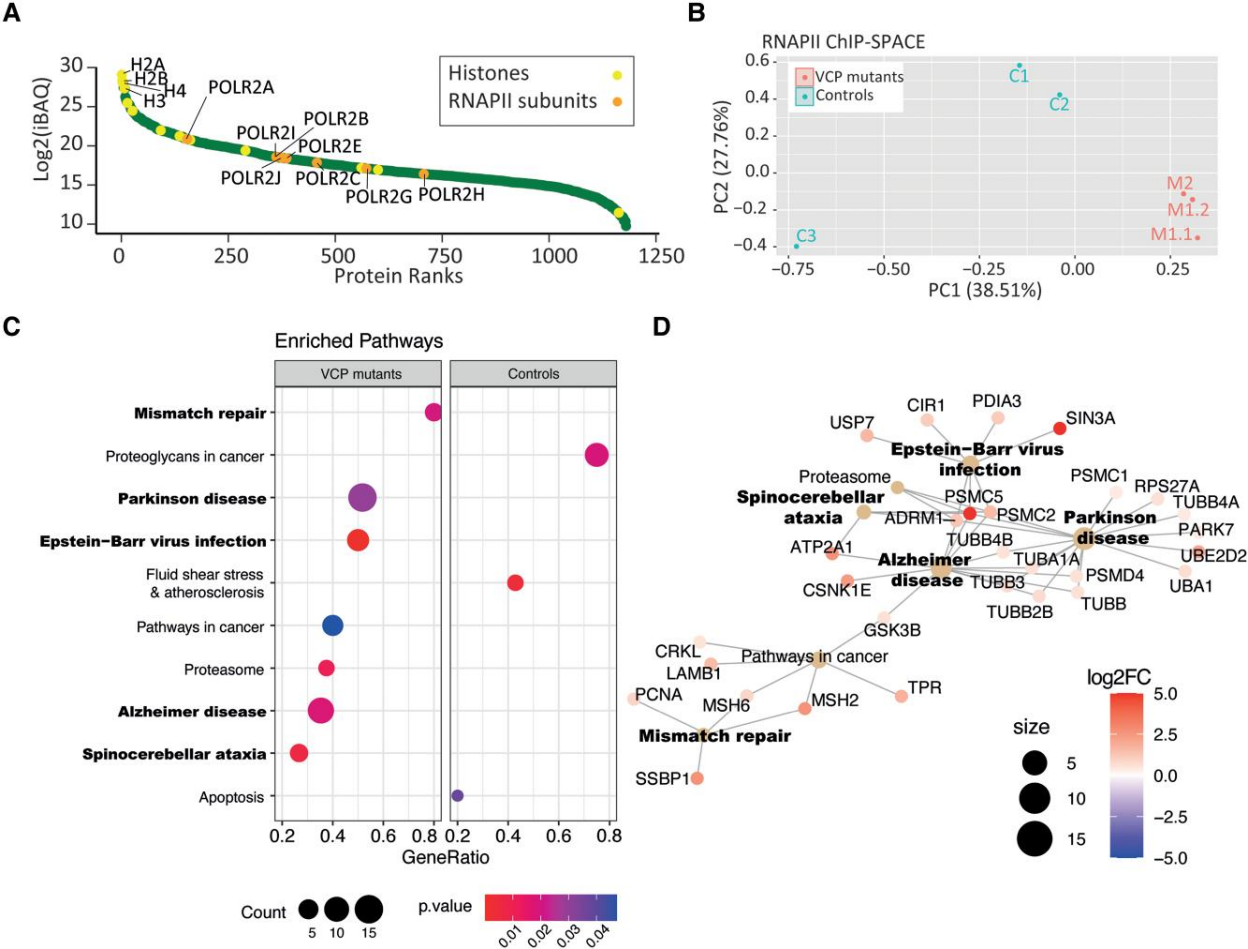
**Co-localized proteins with RNAPII in VCP-mutant and control neural precursors**. (**A**) The RNAPII-associated proteins were sorted based on their iBAQ values (normalized protein intensities). (**B**) Dimensionality reduction of the iBAQ intensities using PCA separates mutant and control cells using PC1. (**C**) KEGG pathway gene set enrichment analysis based on log2-fold-change ratios of the proteins in the mutant and control cells. Node size and colour show number of genes involved in each pathway and *P*-value of enrichment, respectively. (**D**) The enriched pathway and their related genes were shown as a network. Node size and colour show number of genes involved in each pathway and log2-fold-change of mutant/control, respectively.

To understand the potential effect of VCP mutations on RNAPII function in neurodegeneration, we used human iPS cells obtained from ALS patients with heterozygous VCP mutation, which were previously used as cellular models of ALS phenotypes.^15^ The cells were differentiated to neuronal precursors to resemble a model of early events during neurodegeneration (Fig. 1). Two R155C VCP mutant cell lines (M1.1 and M1.2 obtained from one patient) and one R191Q VCP mutant cell line (M2) were compared with three control cell lines from three healthy donors (Supplementary Table 1). We fixed the cells in the plates by adding formaldehyde to crosslink the chromatin-binding proteins to DNA, then sheared chromatin to <1 kb fragments by sonication. We targeted RNAPII by immunoprecipitation to identify the associated proteins. We then used DNA-binding beads (SPACE procedure) to stringently purify chromatin fragments and to remove artificial interactions that are produced during the immunoprecipitation. Following MS, we compared the proteins identified by RNAPII and normal IgG to remove the potential background proteins. After quantifying the proteins by MS, we sorted the proteins based on their iBAQ values to check the quality of chromatin purification. Histones are the most abundant proteins, followed by RNAPII components (Fig. 2A). Comparing iBAQ values separated the control and mutant cells using principal component analysis (PCA; Fig. 2B). Interestingly, KEGG pathway enrichment by gene set enrichment analysis shows that neurodegenerative disorders such as Parkinson’s disease, Alzheimer’s disease and spinocerebellar ataxia are enriched in the mutant cells (Fig. 2C and D). Additionally, we observed the enrichment of mismatch repair and Epstein–Barr virus (EBV) infection in the mutant cells. Increasing evidence has linked neurodegeneration to DNA damage and dysfunctional repair.^24^ Specifically, ALS-associated mutations were shown to affect DNA damage response activation.^25^ As such, DNA damage is accumulated in motor neurons differentiated from patients’ induced pluripotent stem cells and in spinal cord tissues.^26–28^ Recent studies have shown that EBV can induce neuroinflammation during latency and reactivation phases by arising systemic immune response, which leads to demyelination in multiple sclerosis^29^ and neurodegeneration in Parkinson’s disease and Alzheimer’s disease.^30^ The underlying mechanism, however, is not understood, and further mechanistic studies are required to elucidate the role of viral infection in the pathology of ALS. Altogether, this result indicates characterized and novel pathways involved in neurodegeneration and highlights the quality of the data for studying the effect of VCP mutations in ALS.

### ALS-associated proteins differentially bind RNAPII

**Figure 3:**
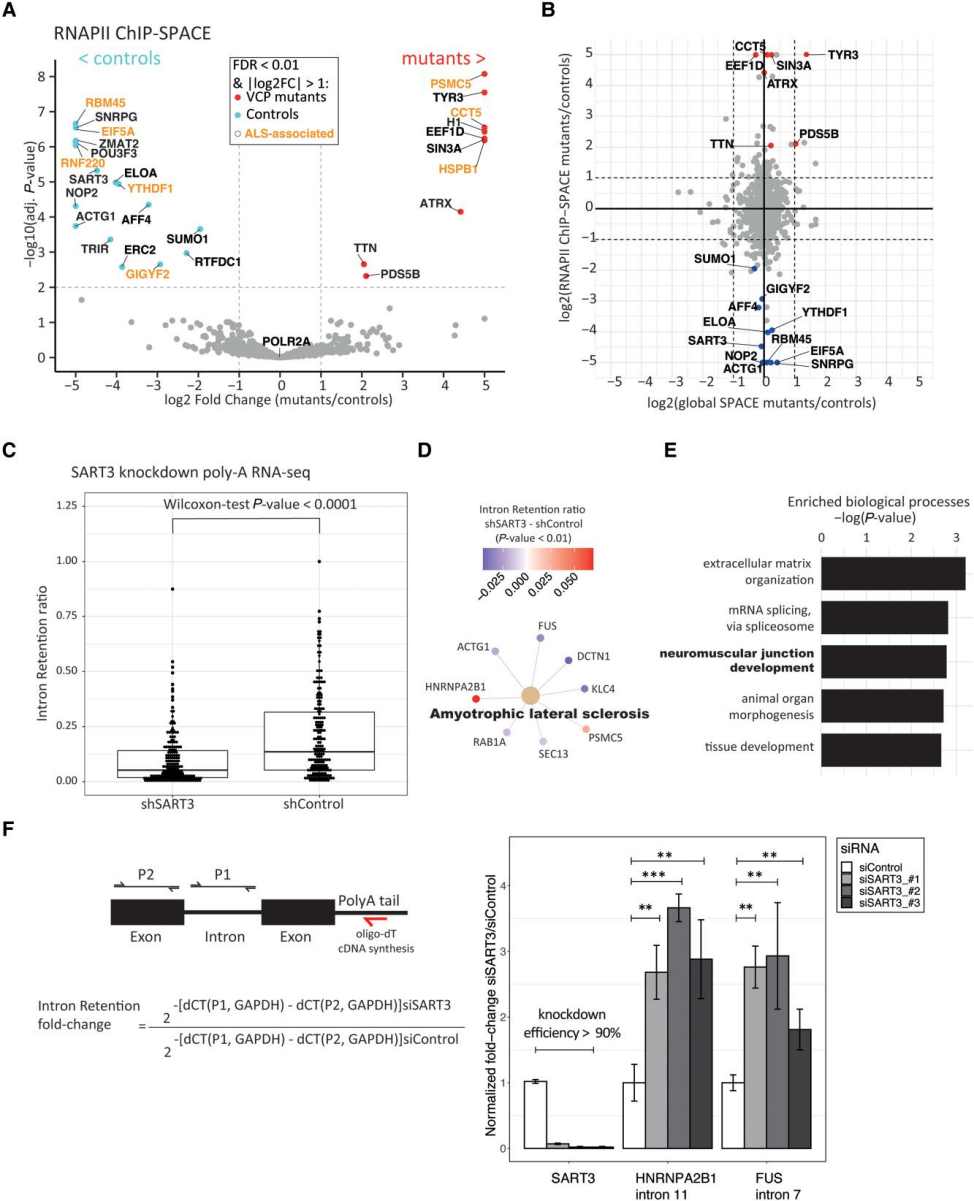
**Statistically significant proteins in RNAPII complex of VCP mutant and control cells**. (**A**) The volcano plot shows the proteins that differentially bind RNAPII in VCP-mutant and control cells with red and blue dots, respectively. The ALS-associated proteins are indicated by the orange colour. (**B**) Global quantification of chromatin-binding proteins and comparison with the RNAPII ChIP-SPACE fold changes. (**C**) We analysed available poly-A RNA-seq data of HEP G2 knockdown of SART3 by using IRfinder to calculate intron retention (IR) ratio for each transcript. The mean of intron retention ratios between SART3 knockdown and control was compared using a paired Wilcoxon-test. The boxes show IQR. The lower and upper whiskers show the 1st quartile − 1.5 × IQR and the 3rd quartile + 1.5 × IQR, respectively. The lines in the box plot show medians. (**D**) KEGG pathway gene set enrichment analysis among the genes with IRfinder *P*-value < 0.01. Node colours show the difference of intron retention ratios (shSART3 − shControl). (**E**) GO enrichment analysis using genes with IRfinder *P*-value < 0.01. The rest of the genes were used as the background. (**F**) The role of SART3 in intron retention of *FUS* and *HNRNPA2B1* in BJ cells was verified by siRNA knockdown and RT-qPCR. BJ cells were transfected by three different siSART3 separately. Each transfection was carried out three times independently. To target mature mRNA, cDNA was synthesized using oligo-dT. The primers were designed to target the introns that were found to be significantly perturbed using IRfinder. Genomic DNA was removed by Turbo DNase treatment and verified by noRT control. The exons of the same genes and GAPDH were used to normalize the expression of the introns. The siSART3s and the siControl were compared using *t*-test to determine significant perturbations: **P-value < 0.01, ***P-value < 0.001.

Using Bayesian-moderated *t*-test and Benjamini–Hochberg procedure to control FDR, we shortlisted the proteins that were statistically significant between the mutant and control groups. Of 1126 RNAPII-associated proteins identified (Supplementary Table 1), 18 were significantly enriched in the VCP mutant cells and 10 were significantly depleted relative to the wild-type controls (FDR < 0.01 and log2-fold-change > 1; Fig. 3A). POLR2A showed log2-fold-change = 0.02, which indicates that immunoprecipitation efficiency was almost equal among the samples. We found important players in neurodegenerative disorders that were highly depleted in the VCP mutant group: RBM45, EIF5A, RNF220, YTHDF1 and GIGYF2. For instance, nuclear and cytoplasmic RBM45 inclusions were reported in neurons and glia of ALS disease, TDP-43 associated frontotemporal lobar degeneration and Alzheimer’s disease.^30^ RBM45 is involved in RNA splicing and spliceosome functions; however, the precise function is not clearly understood. Several previous studies have shown that mutant RBM45 relocates to the cytoplasm and co-aggregates with other RNA binding proteins (RBPs) such as TDP-43 into toxic immunoreactive inclusions.^31–33^

EIF5A is a U6 snRNA-binding protein that plays important roles in neurodevelopment. Interestingly, a lysine residue (K50) in EIF5A is post-translationally modified to hypusine, which regulates TDP-43 accumulation and aggregation.^34^ So far EIF5A is the only identified protein in the cells with hypusine modification, which indicates the importance of this protein in human neurodevelopment.^35^

Besides, RNF220 is an E3-ubiquitin ligase that was highly depleted in mutant cells. Notably, RNF220 poly-ubiquitinates TDP-43 and targets it for proteasomal degradation. It has been reported that RNF220 heterozygote mutant mice develop ALS pathology.^36^ GIGYF2 is an RBP, and heterozygous *Gigyf2*^+/−^ mice develop adult-onset neurodegeneration.^37^ YTHDF1 is a reader of m^6^A-modified mRNAs and is required for axon regeneration in neurons.^38^

Furthermore, AFF4 and ELOA, critical factors in the RNAPII transcription elongation,^39,40^ also showed significant depletion from RNAPII complexes in VCP mutant cells. Thus, our data suggest a possible RNAPII elongation defect in mutant cells.

Conversely, we observed three RBPs that are more abundantly co-localized with RNAPII in mutant cells: CCT5, EEF1D and HSPB1. Remarkably, HSBP1 is a chaperone that preserves the proteins in a folding-competent state, and mutations of HSPB1 affect motor neurons of the peripheral nervous system.^41^ CCT5 is also a chaperonin-containing T-complex (TRiC) component, which controls protein aggregation. In Alzheimer’s disease, the expression of CCT5 is decreased and mutation of CCT5 leads to sensory neuropathy.^42^ Interestingly, EEF1D is not only a translation elongation factor, but the long isoform is also a transcription factor that induces heat-shock–responsive genes.^43^ In summary, chaperones and proteins involved in stress response are co-localized more abundantly with RNAPII in the VCP-mutant cells.

To understand if the identified proteins are globally changed on chromatin or specifically on the RNAPII complex, we performed global SPACE to compare the total amount of chromatin-binding proteins between mutant and wild-type cells. None of the proteins with perturbed co-localization with RNAPII showed any significant changes in their global chromatin binding (Fig. 3B). For example, RBM45 co-purifies with RNAPII 32-fold higher from the control cells than the mutant cells but does not show a significant change in chromatin using global SPACE (fold change = 1.1). Thus, the changes observed in RNAPII-associated factors do not result from global changes in the proteome of chromatin.

Altogether, our data indicate that several pathways, including proteostasis and RNA splicing, are perturbed among the RNAPII-associated proteins in the mutant cells. Several of these factors were already studied individually in the context of ALS disease, thus underscoring the validity of our results. We next sought to verify SART3 as one of the top hits and uncharacterized proteins in the context of ALS disease. As SART3 is involved in recycling the splicing factors,^44^ we hypothesized there is a link between SART3 deficiency and perturbed alternative splicing in ALS.

### SART3 knockdown perturbs splicing of neuromuscular junction development genes

SART3 functions in recycling the splicing factors by promoting the reassembly of U4/U6 snRNP after splicing.^44^ SART3 directly interacts with SNRPG, which is a core component of the spliceosomal U1, U2, U4 and U5 snRNPs. Downregulation of SART3 and a few other splicing factors in the RNAPII complex suggest splicing is perturbed in the mutant cells. To validate this hypothesis, we searched the publicly available data sets and found SART3 knockdown combined with poly-A RNA-seq data using Hep G2 cells.^22^ In fact, aberrant alternative splicing such as intron retention is frequently observed in post-mortem samples of ALS patients.^45,46^ We therefore compared the SART3 knockdown with the control to quantify intron retention events using IRfinder.^23^ In general, we observed lower intron retention in the SART3 knockdown cells (Fig. 3C and Supplementary Table 2). We then analysed the genes with significantly differential intron retention events between the knockdown and control (*P*-value < 0.01) using KEGG pathway gene set enrichment. Surprisingly, the only enriched pathway that we detected was ALS (Fig. 3D). GO analysis of the genes with significantly differential intron retention events indicates that neuromuscular junction development is among the top three overrepresented biological processes (Fig. 3E). Indeed, previous studies have shown that synaptic connections between motor neurons and skeletal muscles are highly disturbed in ALS such that neuromuscular junction damages may occur in the initial stages of the disease.^47^ Furthermore, enrichment of extracellular matrix organization genes such as different collagens points towards other aspects of the VCP mutations that are associated with musculoskeletal disorders.^48^ We sought to further validate the role of SART3 in regulating intron retention in non-cancer cells (BJ cells). To this end, we targeted SART3 using three different siRNAs separately (Fig. 3F). We prepared cDNA using oligo-dT to target mature mRNAs in qPCR. Using the IRfinder results, we designed primers to compare the expression of *HNRNPA2B1* (intron 11) and *FUS* (intron 7) as examples of ALS-associated genes with significant perturbation of intron retention. We normalized the expression of the introns by an exon of the same gene and by GAPDH. As a result, we observed in average 3-fold and 2.5-fold increase in the intron retention of *HNRNPA2B1* (intron 11) and *FUS* (intron 7), respectively (*t*-test *P*-values < 0.01).

Collectively, this result strongly links SART3 deficiency to ALS-associated genes via intron retention events and independently verifies our previous observation that SART3 is downregulated in the RNAPII complex of the VCP-mutant cells.

## Discussion

**Figure 4:**
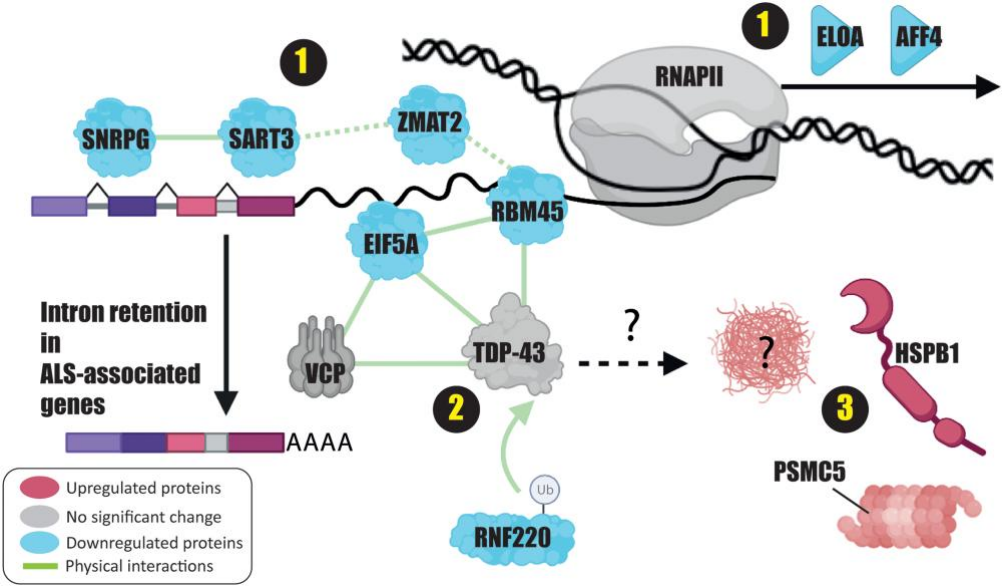
**Three perturbed pathways in the transcriptional machinery of the VCP-mutant cells**. (1) Splicing and transcription machinery: Several proteins involved in mRNA splicing show a significant reduction in the mutant cells. Additionally, lower ELOA and AFF4 affect RNAPII elongation, which also affects alternative splicing. At least, depletion of SART3 impairs intron retention in ALS-associated genes. (2) TDP-43 regulation: RNF220, RBM45 and EIF5A that are regulators of TDP-43 show diminished binding to RNAPII. RNF220 is an E3-ubiquitin ligase that targets TDP-43 for proteasomal degradation. The lack of RNF220 in the RNAPII complex of the mutant cells potentially makes TDP-43 susceptible to accumulation and aggregation close to the transcription machinery. (3) Protein refolding and degradation: The mutant cells recruit heat shock proteins, such as HSPB1 and PSMC5 (a regulating subunit of proteasome) as a defensive mechanism to alleviate protein aggregation. The figure was created with BioRender.com.

VCP mediates ubiquitin-dependent protein extraction from chromatin through the ubiquitin–proteasome system.^2^ Inactivation of VCP leads to protein-induced chromatin stress and aggregation, which has been studied in the context of DNA repair and replication.^2^ Our data provide a model to understand how VCP mutations affect transcriptional machinery in an ALS model system. Our findings reveal at least three pathways that are perturbed in the mutant cells (Fig. 4). The first pathway is related to transcription and splicing machinery, which are highly interconnected. Our results indicate decreased binding of RNAPII elongation factors AFF4 and ELOA (Elongin A) in the mutant cells. AFF4 is a core component of the super elongation complex and acts as a scaffold protein for other proteins that together are required for increasing the catalytic rate of RNAPII.^45^ ELOA is the largest subunit of the elongin complex, which stimulates RNAPII elongation.^49^ ELOA regulates the transition of paused RNAPII to the elongation state.^50^ While an optimal rate of transcription elongation is needed for proper pre-mRNA splicing,^51^ combined deficiency of AFF4 and ELOA might indicate inconsistent RNAPII elongation in the mutant cells, which leads to aberrant splicing. Furthermore, several RBPs involved in mRNA processing and splicing such as ZMAT2, RBM45, SNRPG and SART3 are decreased in the proximity of RNAPII in the VCP-mutant cells. Interestingly, SART3 knockdown leads to perturbation of intron retention in ALS-associated genes. Although this experiment was carried out in HEP G2 cells, ALS pathway and neuromuscular junction development genes show significant enrichment. Using HEP G2 cells, some genes, such as *FUS*, show lower intron retention in SART3 knockdown in comparison to the control, whereas some others, such as *HNRNPA2B1*, have higher intron retention. We verified the effect of SART3 knockdown in BJ cells as a non-cancer cell line. Both *HNRNPA2B1* and *FUS* show significantly increased intron retention in BJ cells upon SART3 knockdown. While the precise effect of SART3 knockdown on splicing could be cell-type dependent, in general, transcript isoforms with intron retention trigger non-sense mediated decay, which leads to the downregulation of the proteins.^52^ Thus, perturbation of intron retention in ALS-associated genes, such as *FUS*, probably deregulates their balance of translation and proteostasis, which has been linked to the ALS disease.^53^

Second, we found RBM45, EIF5A and RNF220 as direct interactors and regulators of TDP-43 that are dramatically reduced in the mutant cells. TDP-43 aggregation is observed in many neurodegenerative disorders,^54^ including >97% of sporadic and familial ALS patients.^55^ However, TDP-43 is mutated in 1–4% of the cases.^56^ This indicates that regulators of TDP-43 are affected in ALS disease, thus allowing TDP-43 to accumulate and aggregate. Recently, RNF220 was introduced as a regulator of TDP-43 that targets it for proteasome degradation by polyubiquitination.^36^ RNF220 is required for the function and development of the cerebellum and central motor neurons in mice.^57^ Interestingly, *Rnf220*^+/–^ mice accumulate TDP-43 in their cytoplasm. The mice also develop phenotypes similar to ALS such as hindlimb paralysis, muscle wasting and neuronal loss.^36^ We speculate that reduced RNF220 in RNAPII complexes of VCP-mutant cells could be an early step that leads to TDP-43 aggregation.

The third pathway is upregulated in the mutant cells, as heat shock proteins such as HSPB1 and CCT5 show increased binding to the RNAPII complex in the mutant cells. Mutations of HSPB1 have been reported in Charcot–Marie–Tooth neuropathy, distal hereditary motor neuropathies and ALS.^58^ Recent findings revealed HSPB1 as a regulator of TDP-43 phase separation. HSPB1 co-localizes with TDP-43 droplets, prevents TDP-43 assembly into fibrils and is essential for the disassembly of stress-induced TDP-43 droplets.^59^ We also observed increased binding of PSMC5, a subunit of 19 s proteasome, which has ATPase activity to unfold the ubiquitinated proteins and has chaperone-like activity.^5^ The increased binding of heat shock proteins and chaperones, specially HSPB1, provides a protective mechanism against protein aggregation and misfolding.

Taken together, our study sheds light on the disrupted pathways in the transcriptional machinery in an ALS model system with VCP mutations. This situation could potentially lead to proteinopathy and aberrant alternative splicing, which is observed in neurodegeneration. In the future, RNAPII ChIP-SPACE would be a valuable approach for further analyses of additional cell types and samples from ALS patients with mutations in multiple genes or sporadic ALS cases to gain a more comprehensive insight into the pathological mechanism of the disease.

At present, 8 of 28 differential proteins identified by our ChIP-SPACE approach have been shown by previous studies to be associated with neurodegeneration (Fig. 3A). We functionally validated the role of SART3 in intron retention of ALS-associated genes (Fig. 3C–F). We also aimed to validate ChIP-SPACE results by western blot. Despite our best efforts we could not detect any bands by western blot, not even RNAPII, which is the target of the ChIP-SPACE. While western blot cannot be used as the validation of this approach, it remains a limitation of this study to further validate the differential proteins by other orthogonal methods.

## Supplementary Material

awad046_Supplementary_Data
