# Supplementary material for: RNA polymerase II-associated proteins reveal pathways affected in neural precursors with VCP mutation
Source: Brain. Author manuscript; Available in PMC 2023 Jul 12. (PMC7614746; doi:10.1093/brain/awad046)
Supplement: Supplementary file 1 [file EMS177491-supplement-Supplementary_material.docx]

# Supplemental material

Supplementary Table 1. RNAP2- and global-SPACE datasets.

- Spreadsheet 1: RNAP2-SPACE

- Spreadsheet 2: RNAP2-SPACE versus global-SPACE

- Spreadsheet 3: RNAP2-SPACE raw values

- Spreadsheet 4: global-SPACE raw values

Supplementary Table 2. SART3 knockdown and intron retentions

- IRfinder

- GO enrichment
